# Enhancement of microwave absorption performance of porous carbon induced by Ce (CO_3_) OH

**DOI:** 10.3389/fchem.2022.1100111

**Published:** 2023-01-09

**Authors:** Jijun Wang, Yuhua Chen, Yaxing Wei, Yan Li, Fangyuan Li, Bingzhen Li, Qingqing Wu, Jinlong Zhao

**Affiliations:** ^1^ Chinese People’s Liberation Army, Beijing, China; ^2^ Institute of Defense Engineering, Academy of Military Sciences, Beijing, China

**Keywords:** microwave absorbers, impedance matching, rare earth compounds (Ce (CO3) OH), Ce (CO3) OH/C composite, synergistic effect

## Abstract

In recent years, electromagnetic pollution has become more and more serious, resulting in a very negative impact on people’s health. Therefore, it is important to develop efficient microwave absorbers to reduce electromagnetic pollution. Here, we construct a novel absorbing material of the polymer gel-derived porous carbon decorated by rare earth compounds (Ce (CO_3_) OH). When the thickness is 2.2 mm, the composite exhibits excellent microwave absorption performance with the optimal RL_min_ value and EAB reached up to −47.67 dB and 5.52 GHz, respectively, covering the Ku band. The high-efficiency microwave absorption is mainly attributed to the synergistic effect of dipole polarization, defect polarization and interfacial polarization. This work not only provides a new view for designing superior absorber materials, but also lay a foundation for their real applications.

## 1 Introduction

With the widespread use of electronic equipment in our daily lives, microwave absorbers have become totally essential to protect human health from serious electromagnetic pollution and improve the apparatus’ communication signal quality (Xu et al., 2019; Zhang et al., 2020; Wang et al., 2021; Nan et al., 2022). So far, much effort have been devoted into the development of target absorbers and the core requirements were identified as light weight, strong absorption, thin layer and broad bandwidth (Phang et al., 2008; Wan et al., 2015; Mo et al., 2019). To achieve these goals, various nano-structural materials have been reported, including magnetic metal oxides (Liu et al., 2013; Zhou et al., 2015), MXene (Han et al., 2017; Huang et al., 2022a), metal alloy (Lutsev and Shutkevich, 2016; Xu et al., 2018; Huang et al., 2022b) and carbonaceous substances (Wu et al., 2021; Wu et al., 2022), etc. Among them, carbon-based nanomaterials derived from gel structure attracted much attention as a result of low density, abundant porosity and adjustable dielectric property (Wang et al., 2022a). By using agar gel containing NiCO_3_ as precursors, Guo et al. synthesized a Ni/C composite consisting of three-dimensional carbon structure with accommodated nickel nanoparticles, which showed an excellent absorbing capability with a broad bandwidth in the range of 13.2 GHz–18 GHz. (Xie et al., 2018). Sun et al. proposed different magnetic metals and porous carbon composite structure on the basis of sol-gel method and subsequent *in situ* pyrolysis process. The prepared composites provided multiple loss pathway of electromagnetic wave and the minimum value of reflection loss was −33.5 dB at 7.7 GHz (Shen et al., 2022). Combining a typical sol-gel approach with supercritical drying and carbonization process, Yang et al. reported a resorcinol-formaldehyde based carbon aerogel with pore structure and good impedance matching. The wide effective bandwidth was estimated to be 4.5 GHz and minimum reflection loss value was determined to be −37.5 dB (Wang et al., 2022b). It follows that further improvement of absorbing performance from gel-derived carbonaceous materials is still limited by the narrow effective bandwidth, which may hinder their practical applications.

In recent, optimization of impedance matching has gradually been proved to expand the absorption bandwidth, and then increase electromagnetic wave attenuation performance (Rehman et al., 2019; Qiao et al., 2020; Jin et al., 2022). Several strategies have been proposed to regulate the dielectric properties of carbon materials, realizing the broadband and strong absorption (Wang et al., 2022c; He et al., 2022; Xu et al., 2022). For example, to reach a minimum reflection loss of −61.2 dB and an effective absorption bandwidth covering 5.2 GHz, Zhu et al. constructed a tubular structured carbon nanofibers coated with titanium dioxide layers, which showed the wave-transparent behavior to change the complex permittivity of carbonaceous constituent and facilitate the composites’ impedance matching (Kang et al., 2020). Similarly, Che et al. optimized the conductivity by anchoring the carbon nanotubes on TiO_2_ nanospheres or designing a multiple structure of vesicle-like-shell TiO_2_@carbon *via* the pyrolyzing the bimetallic zeolitic imidazolate framework encapsulating TiO_2_ nanoparticles or metal-polydopamine-coated TiO_2_, respectively. The obtained absorption performance presented a reflection loss of −44.0 dB and an effective absorption bandwidth of 5.4 GHz at the thickness of 2.0 mm (Ding et al., 2020). To improve its poor impedance-matching characteristic, Zhang et al. modified the ordered mesoporous carbon with silica (OMC-5@SiO_2_) through a self-assemble method and heat treatment process. With the impedance ratio around 1 from 8.56 GHz to 13.2 GHz, the OMC-5@SiO_2_ reached an effective absorption bandwidth of 4.8 GHz and the minimum reflection value of −40.7 dB at 10.8 GHz (Zhou et al., 2020). Nevertheless, the reported strategies are still limited, and new approaches are expected.

In this work, a new composite consisting of the polymer gel-derived porous carbon decorated by rare earth compounds [Ce (CO_3_) OH] was designed and synthesized. Compared to pure carbon materials, the prepared composites [Ce (CO_3_) OH/C] presented the significant higher pyrrolic N and 1.02 I_D_/I_G_ value. The electromagnetic parameters analysis showed that the modified composites’ conductivity occurred downshift with the filling ratio of 10% and the thickness of 1.85 mm. For the electromagnetic wave absorption, by changing the absorbers’ thickness to 2.2 mm, the optimal RL_min_ value and EAB reached up to −47.67 dB and 5.52 GHz, respectively, covering the Ku band. It is believed that the results reported in this work will not only provide a new thought for controlling the impedance matching for carbon-based materials, but also lay a foundation for their real applications.

## 2 Experimental section

### 2.1 Materials

The P123 (PEO-PPO-PEO) material was purchased from sigma-Aldrich. The hexamethylenetetetramine (C_6_H_12_N_4_) and hydrochloric acid (HCl) were bought from Sinopharm Group. The 2,4-dihydroxybenzoic acid (C_7_H_6_O_4_) and cerium nitrate hexahydrate (Ce (NO_3_)_2_·6H_2_O) were purchased from Aladdin Reagent. The ethylenediamine was bought from General Reagent. All chemicals were utilized without further purification.

### 2.2 Preparation of polymer gel

Firstly, 3.56 g P123 was dissolved in 60 ml deionized water and sonicated for 30 min. Then, 0.936 g hexamethylenetetetramine, 3.115 g 2,4-dihydroxybenzoic acid and 415 μl ethylenediamine were added into the above solution, sonicated and dispersed, and transferred to a 100 Teflon-lined stainless autoclave and heatd at 130°C for 4 h. Finally, orange-red product was filtered and washed, and dried in a freeze dryer for 24 h.

### 2.3 Preparation of carbon materials

First, 1 g of polymer gel and 4 g of calcium carbonate nanoparticles were well ground and calcined in a tube furnace under nitrogen atmosphere at 900°C for 2 h. Then, the calcination product was immersed in 40 ml of 3 M HCl and stirred for 1 h to remove the generated calcium oxide. Finally, the product was filtered, washed with deionized water until neutral, and dried overnight at 70°C under vacuum.

### 2.4 Preparation of porous carbon loaded cerium nanomaterials (Ce/PC)

First, 100 mg of the above carbon materials and 200 mg of Ce (NO_3_)_2_·6H_2_O were added to 30 ml deionized water, sonicated for 2 h and stirred for 24 h, and then dried under vacuum for 24 h. Finally, the product was calcined at 1,000°C for 1 h under N_2_ with a heating rate of 5°C min^−1^. In addition, the blank sample PC was obtained by direct pyrolysis of a carbon material without adsorbed Ce.

### 2.5 Characterization and tests

The crystal structure was measured by X-ray diffraction (Bruker, D8 Advance). The micro-morphology and micro-structure were observed by scanning electron microscope (SEM) (Tescan MIRA3 LMU) and transmission electron microscopy (TEM) (JEOL JEM-2100F). Chemical composition was studied by X-ray photoelectron spectroscopy (XPS) on a K-Alpha 1063 X-ray photoelectron spectrometer. Raman tests were recorded on Renishaw Raman spectroscopy. The microwave absorption (MA) performance was investigated by a microwave vector network analyzer (VNA, Agilent N5230A).

## 3 Results and discussion

### 3.1 Structural and morphological analysis

**FIGURE 1:**
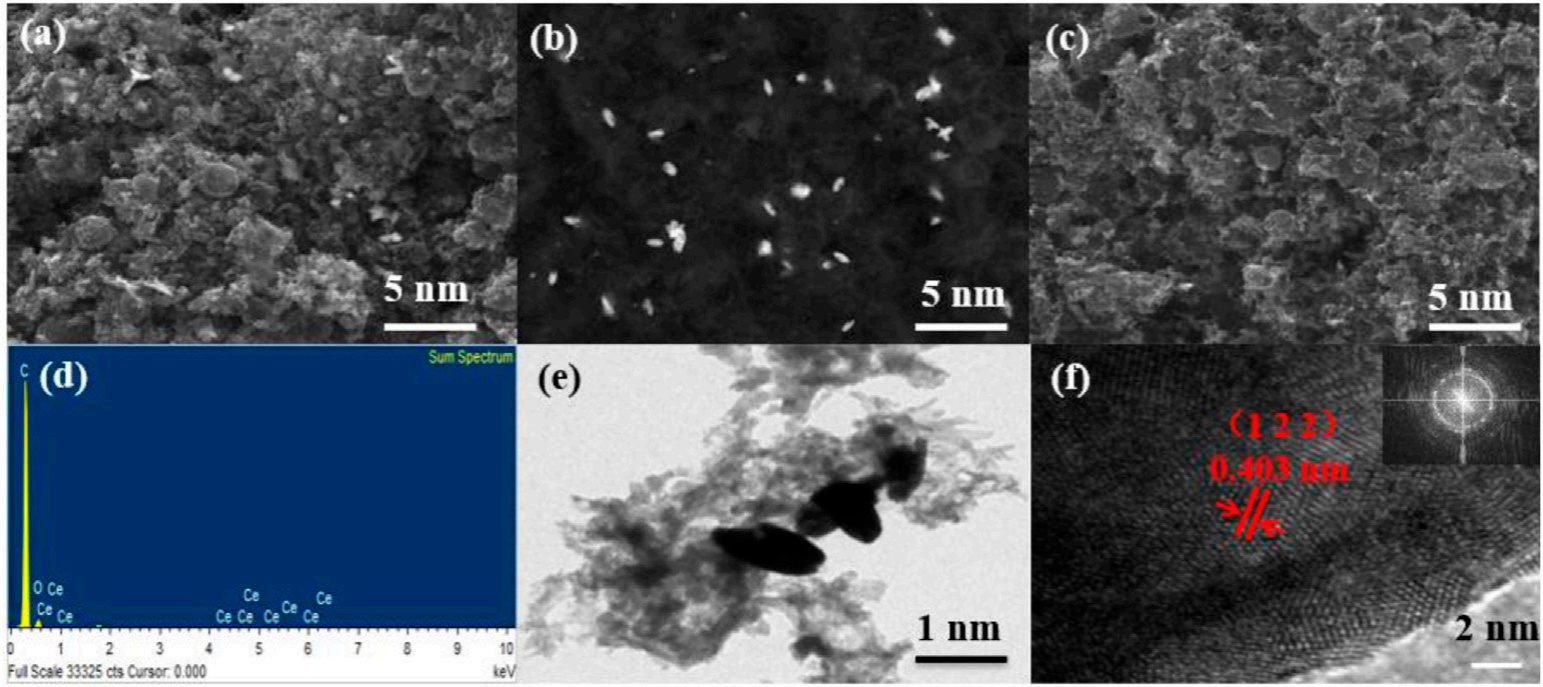
**(A)** Bright-field SEM picture for Ce/PC; **(B)** Dark field SEM picture for Ce/PC; **(C)** Bright-field SEM picture for PC; **(D)** EDS picture of Ce/PC; **(E)** TEM image of Ce/PC; **(F)** HR-TEM and SAED images of Ce/PC.

The surface morphology and elemental composition of the prepared samples were analyzed by scanning electron microscopy (SEM). As shown in Figures 1(A–C), there is no significant difference in the micro-morphology between Ce/PC and PC samples, which are composed of irregular bulk particles. Ce grows in porous carbon to form Ce/PC sample, which is confirmed by EDS pattern of Ce/PC sample (Figure 1D). The TEM image (Figure 1E) show granular Ce growing on a porous carbon skeleton substrate. Additionally, the related HR-TEM (Figure 1F) image for Ce/PC shows clear lattice space of 0.403 nm, which is attributed to (1 2 2) plane of Ce (CO_3_) OH, further demonstrating the successful addition of Ce. The selected area electron diffraction (SAED) exhibits a ring-shape pattern corresponding to the Ce (CO_3_) OH.

**FIGURE 2:**
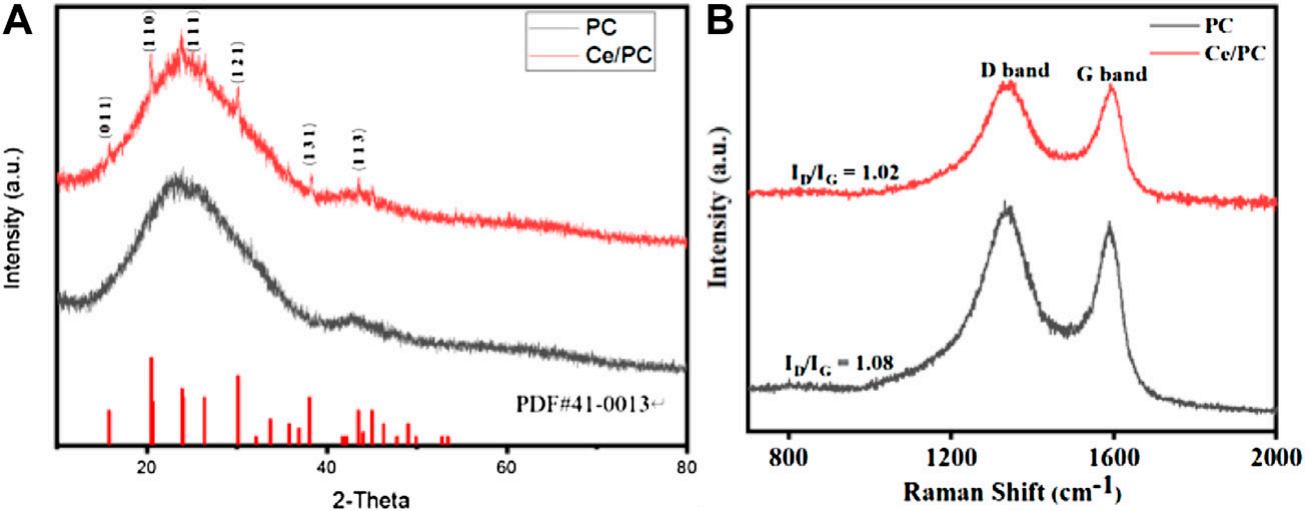
**(A)** XRD patterns and **(B)** Raman spectra for Ce/PC and PC.

**FIGURE 3:**
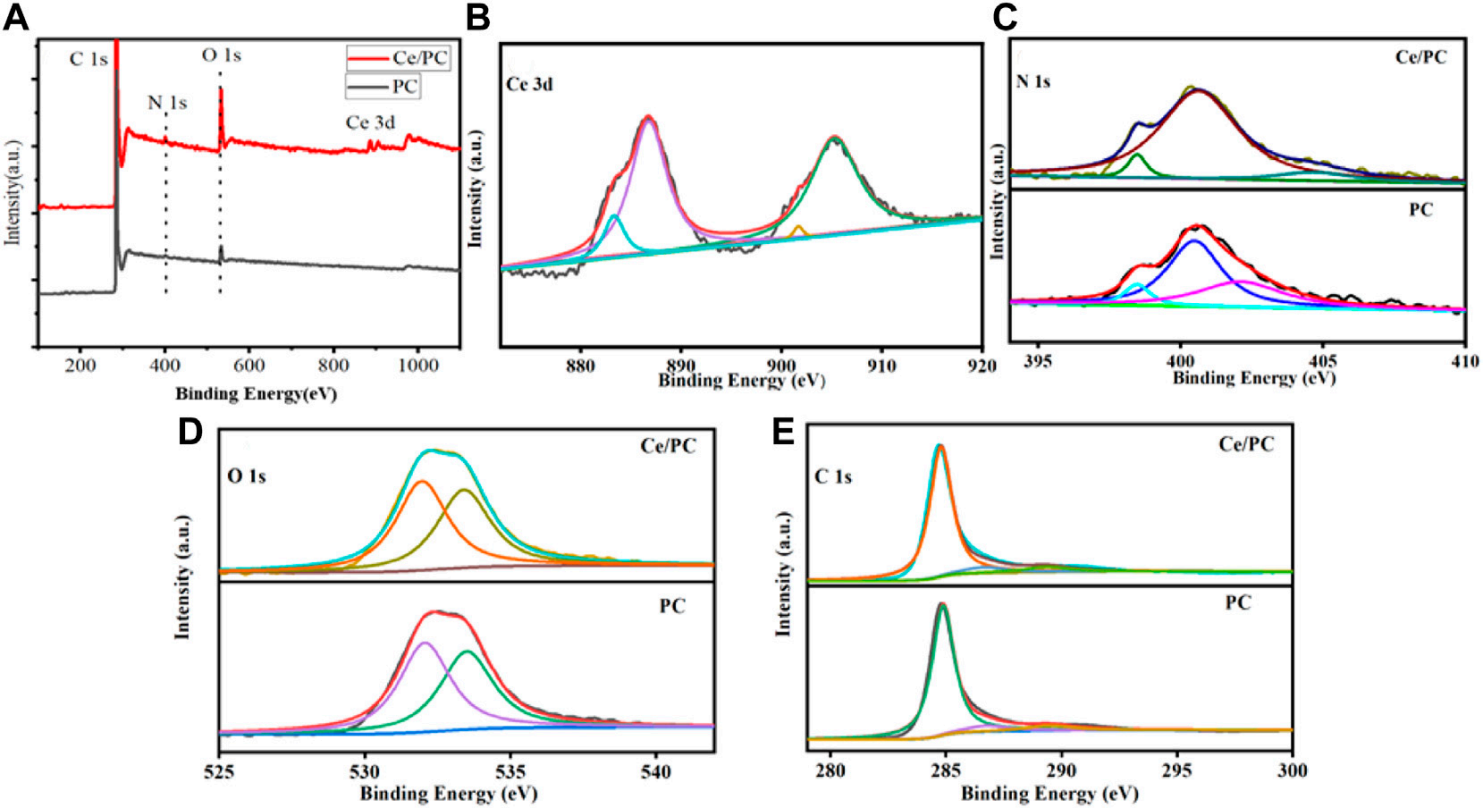
**(A)** XPS survey spectra for Ce/PC and PC; **(B)** Ce 3d XPS spectrum for Ce/PC; **(C)** N 1s XPS spectra, **(D)** O 1s XPS spectra and **(E)** C 1s XPS spectra for Ce/PC and PC.

It can be seen from the X-ray powder diffraction (XRD) pattern in Figure 2A that both Ce/PC and PC samples have two broad diffraction peaks at 26.5°C and 43.3°C, which are attributed to (0 0 2) and (1 0 1) planes of carbon. The characteristic peaks of Ce/PC is the same as that of the standard comparison card PDF # 41-0013, which proves that Ce in Ce/PC sample exists in the form of Ce (CO_3_) OH. Normally, the degree of graphitization of a material can be characterized by Raman spectroscopy. From Figure 2B, both Ce and Ce/PC exhibit two peaks located at around 1,333 cm^−1^ (D band) and 1,587 cm^−1^ (G band) The D band signifies the sp^3^ defects or disorder, and G band indicates the sp^2^ hybridization. The I_D_/I_G_ is usually used to reflect the degree of disorder (Shu et al., 2018a; Wu et al., 2019). It can be seen from Figure 3B that the I_D_/I_G_ of the prepared material decreases slightly with the doping of Ce, which indicates that the degree of defect or disorder is reduced in Ce/PC sample.

The chemical composition of PC and Ce/PC sample was determined by X-ray photoelectron spectroscopy (XPS). The peaks of Cand Ce elements were found in the XPS survey spectrum of Ce/PC (Figure 3A), further confirming the presence of these elements in the Ce/PC sample. In Ce 3d spectra (Figure 3B), Ce/PC sample shows four characteristic peaks. The Ce 3d_3/2_ is marked as U and U′, and Ce 3d_5/2_ is marked as V and V′. The characteristic peaks U′ and V represent Ce^3+^, and the characteristic peaks U and V′ represent Ce^4+^ (Wu et al., 2020).Therefore, Ce^4+^ and Ce^3+^ coexist in Ce-PC, indicating the presence of oxygen vacancies. The electric conductivity increases with the increase of oxygen vacancy defects (Shu et al., 2018a; Wu et al., 2019). The enhanced electric conductivity is beneficial to enhancing the conduction loss and charge polarization relaxation of Ce-PC sample (Chen et al., 2020; Zhao et al., 2020). The N 1s spectra (Figure 3C) demonstrated the presence of pyridinic-N (398.4 eV), graphitic-N (400.5 eV) and oxide-N (402.1 eV,404.7 eV) in PC and Ce/PC samples (Yang et al., 2020). From Figure 3D, the O 1s spectra can be fitted into two peaks of -COOH (531.9 eV) and -OH (533.4 eV). (Soren et al., 2016). As shown in Figure 3E, the peaks of C 1s at 289.3 eV, 286.9 eV and 284.8 eV can be assigned to O-C=O, C-O and C=N bonds, respectively.

### 3.2 Microwave absorption properties

The microwave absorption properties of microwave absorbing materials can be evaluated by reflection loss (RL). According to the transmission line theory, the RL is calculated by the following formula (Wang et al., 2019a; Liu et al., 2019; Luo et al., 2020):

$$R_{L}=20\mathrm{lg}\frac{\left|Z_{in}-Z_{0}\right|}{\left|Z_{in}+Z_{0}\right|}$$ (1)

$${\;Z}_{in}=\sqrt{\frac{\mu_{r}}{\varepsilon_{r}}}\tanh\left[j\frac{2\pi }{c}\sqrt{\mu_{r}\varepsilon_{r}}fd\right]$$ (2)

Herein Z_in_ is the input impedance of absorber, Z_0_ is the impedance of free space, $\varepsilon_{r}$ is the relative complex permittivity, $\mu_{r}$ is the relative complex permeability, d is the thickness of the absorber, c is the velocity of light in free space, and f is the frequency.

**FIGURE 4:**
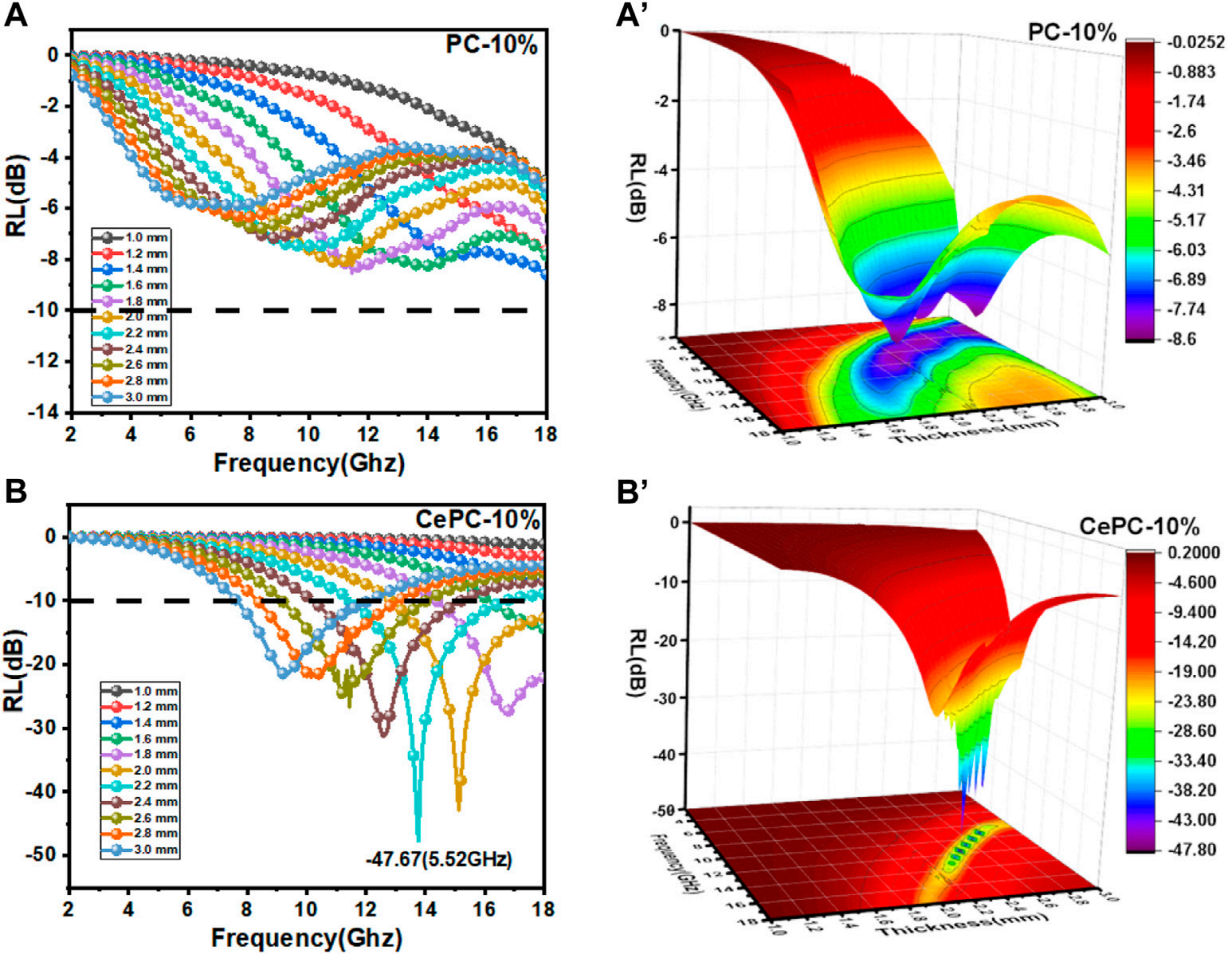
Frequency dependence of reflection loss with different thicknesses and 3D plots of reflection loss: **(A)** and **(A')** PC, **(B)** and **(B')** Ce/PC.

As described in Figures 4A, 4A', the RL_min_ of PC samples is less than 10 dB, indicating that pure PC materials have weak microwave absorption properties. From Figures 4B, B', compared with PC samples, the RL_min_ of Ce/PC samples at 13.76 GHz is −47.67 dB, showing significantly improved microwave absorption properties, the corresponding thickness is 2.2 mm, and the effective absorption bandwidth (EAB, RL < −10 dB) can reach 5.52 GHz. In addition, more than 10 dB can be achieved by adjusting the matching thickness from 1 mm to 3 mm, covering almost the entire Ku and X-band.

**FIGURE 5:**
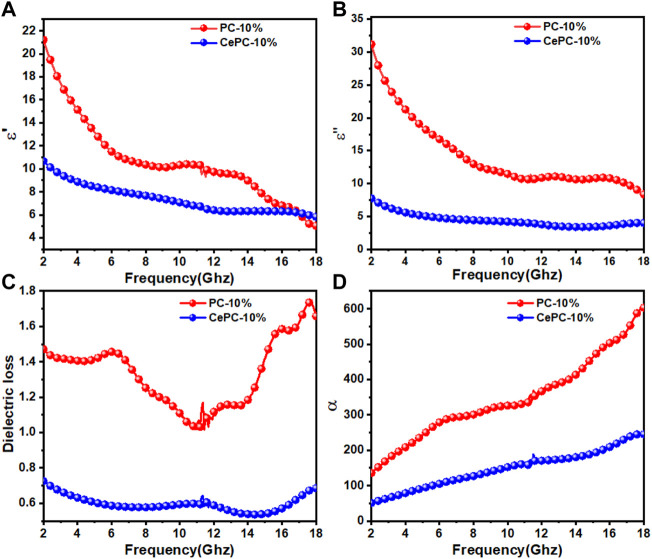
Electromagnetic parameters of PC and Ce/PC: **(A)** the real part and **(B)** the imaginary part of complex permittivity, **(C)** dielectric loss and **(D)** attenuation constant α.

Generally, the electromagnetic parameters ( $\varepsilon^{\prime }$ , $\varepsilon^{\prime \prime }$ , $\mu^{\prime }$ , $\mu^{\prime \prime }$ ) are vitally important to determine the microwave absorption properties of absorbers (Shu et al., 2018b; Shu et al., 2019a; Shu et al., 2019b). The real permittivity ( $\varepsilon^{\prime }$ ) and real permeability ( $\mu^{\prime }$ ) represent the storage ability of electric and magnetic field energies, whereas the imaginary permittivity ( $\varepsilon^{\prime \prime }$ ) and imaginary permeability ( $\mu^{\prime \prime }$ ) indicate the dissipation capacity of electric and magnetic field energies, respectively (Shu et al., 2018b; Shu et al., 2019a; Shu et al., 2019b).Since the material we have prepared is almost non-magnetic, only its complex permittivity is discussed here. It can be seen from Figure 5A that the $\varepsilon^{\prime }$ of all samples decreases with the increase of frequency, showing a dispersion behavior, which is beneficial to the attenuation of microwave energy (Quan et al., 2017). Among them, the $\varepsilon^{\prime }$ of PC and CePC samples decreased from 21.21 to 5.04, 10.67 to 5.83, respectively. The results show that the addition of Ce (CO_3_) OH can inhibit the high dielectric behavior of PC materials and improve the properties of the materials. As shown in Figure 5B, with the increase of frequency, $\varepsilon^{\prime }$ and $\varepsilon^{\prime \prime }$ show a similar trend of change. According to the free electron theory, $\varepsilon^{\prime \prime }$ increases with the increase of electrical conductivity. However, pure Ce is an electrical insulator. On the contrary, the introduction of it suppresses the conductive behavior of PC materials, so the $\varepsilon^{\prime \prime }$ of Ce/PC is lower than that of PC materials.We further analyzed the dielectric loss tangent (tanδԑ = $\frac{\;{ԑ}^{\prime \prime }}{ԑ՛}$ ). From Figure 5C, the tanδԑ value of pure PC material is higher than that of CePC, thanks to the high $\varepsilon^{\prime \prime }$ of PC. However, due to the introduction of Ce (CO_3_) OH, the tanδԑ value of Ce/PC material is in the range of 0.5–0.8, which weakens the dielectric loss ability of PC to some extent.

The attenuation ability of electromagnetic wave is usually reflected by the attenuation constant ( α ), which can be expressed as follows (Shu et al., 2019b; Wang et al., 2019b; Shu et al., 2019c; Zhang et al., 2019):

$$\alpha =\frac{\sqrt{2}\pi f}{c}\times \sqrt{\left(\mu^{\prime \prime }\varepsilon^{\prime \prime }-\mu^{\prime }\varepsilon \prime \right)+\sqrt{{\left(\mu^{\prime \prime }\varepsilon^{\prime \prime }-\mu^{\prime }\varepsilon \prime \right)}^{2}+{\left(\mu^{\prime \prime }\varepsilon^{\prime }+\mu^{\prime }\varepsilon \prime \prime \right)}^{2}}}$$ (3)

Figure 5D shows the frequency dependence of the attenuation constant an of the prepared samples. As can be seen from Figure 5D, compared with PC materials, Ce/PC shows relatively weak electromagnetic wave attenuation ability. The change of attenuation capacity mainly comes from the change of dielectric loss. However, PC does not show the best microwave absorption performance, as shown in Figure 4. Therefore, the impedance matching characteristics need to be further considered.

**FIGURE 6:**
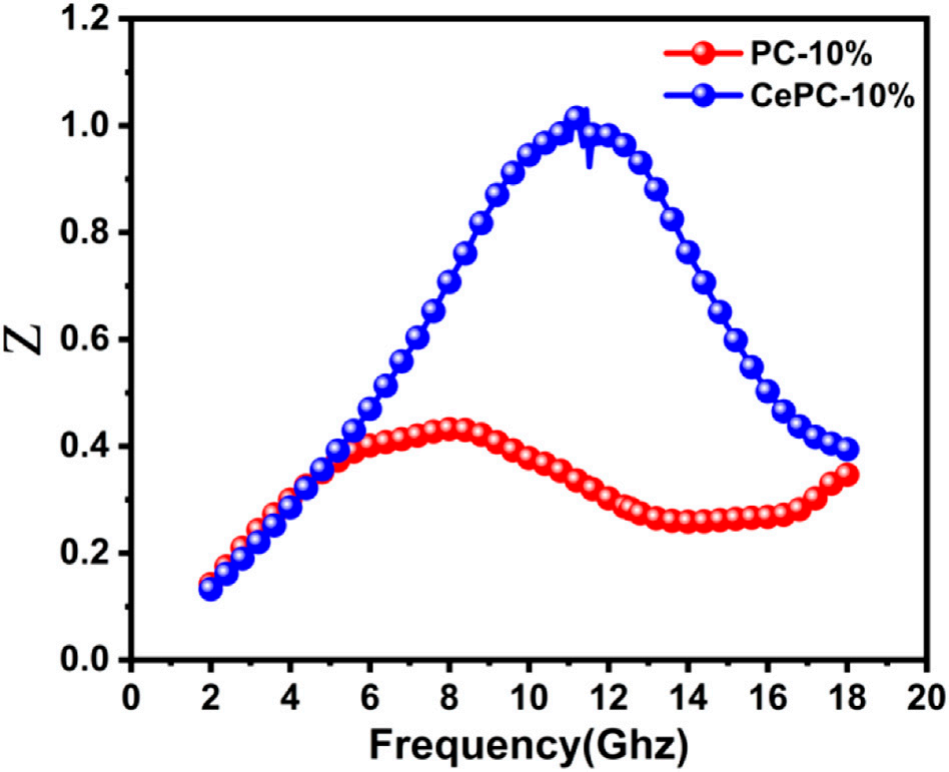
Frequency dependence of normalized impedance matching (Z) for the samples of PC and Ce/PC.

As shown in Figure 6, the Z value of pure PC material is far from 1, which indicates that the impedance matching is poor. The Ce/PC material is closer to the optimal impedance matching line (Z = 1), which indicates that the impedance matching has been greatly improved. Due to the realization of the best impedance matching, most of the incident microwaves can enter the material (Liu et al., 2017a; Wang et al., 2019c). At the same time, the moderate electromagnetic attenuation ability can effectively convert electromagnetic energy into thermal energy (Shu et al., 2019a; Wang et al., 2019c; Zhang et al., 2019). Therefore, Ce/PC shows better microwave absorption properties.

According to Debye theory, dielectric loss includes conduction loss and polarization loss (Liu et al., 2017b). $\varepsilon^{\prime }$ and $\varepsilon^{\prime \prime }$ follow the equation (Liu et al., 2017b; Shu et al., 2018b; Shu et al., 2019b):

$$\left(\varepsilon^{\prime }-\frac{\varepsilon_{\infty }+\varepsilon_{s}}{2}\right)+{\left(\varepsilon^{\prime \prime }\right)}^{2}={\left(\frac{\varepsilon_{\infty }-\varepsilon_{s}}{2}\right)}^{2}$$ (4)

**FIGURE 7:**
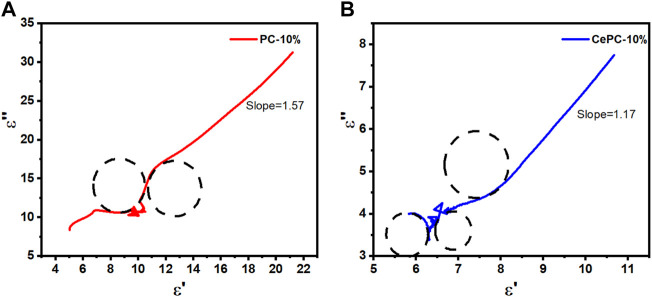
Cole‒Cole semicircle ( $\varepsilon^{\prime \prime }$ ∼ $\varepsilon^{\prime }$ ) curves of the samples: **(A)** PC, **(B)** Ce/PC.

Herein,ԑ_s_, $\varepsilon_{\infty }$ , $\varepsilon^{\prime }$ and $\varepsilon^{\prime \prime }$ are the static permittivity, relative dielectric permittivity at high-frequency limit, real part and imaginary part of permittivity, respectively (Liu et al., 2017b; Shu et al., 2018b; Shu et al., 2019b). Based on Eq. 4, the curve of ( $\varepsilon^{\prime \prime }$ ∼ $\varepsilon^{\prime }$ ) should be a single semicircle, which is known as Cole-Cole semicircle (Liu et al., 2017b; Shu et al., 2018b; Shu et al., 2019b). Each semicircle represents a Debye relaxation process (Liu et al., 2017b; Shu et al., 2018b; Shu et al., 2019b). Figures 7A,B shows the Cole-Cole diagram of sample PC and Ce/PC. From Figures 7A,B, PC and Ce/PC show two and three semicircles, respectively, indicating that Ce/PC materials have more relaxation processes. This is mainly due to the increase of internal defects caused by the introduction of Ce (CO_3_) OH, which leads to the increase of polarization relaxation of defect dipoles, which can be obtained by further analysis of XPS and Raman spectra. In addition, it can be observed that the semicircle is deformed to a certain extent, and the Cole-Cole diagram has a part of the tail straight line region, which indicates that Debye relaxation is not the only mechanism of dielectric loss, and other mechanisms such as conductive loss may be the cause of microwave absorption (Shu et al., 2018b; Shu et al., 2018c). By fitting the straight line part of the Cole-Cole diagram, the slopes are 1.57 and 1.17 respectively, which indicates that the conductive loss plays a more important role in PC materials.

We analyzed the possible microwave absorption mechanism of Ce (CO_3_) OH/C nanocomposites. First of all, under the action of alternating electromagnetic field, the residual groups (- COOH and-OH) and structural defects on the surface or edge of porous carbon will cause dipole polarization and defect polarization (Suresh et al., 2013; Shu et al., 2018a), respectively. At the same time, Ce (CO_3_) OH nanoparticles can also be used as polarization centers to further enhance dipole polarization relaxation (Wu et al., 2019). Secondly, a large number of heterogeneous interfaces between paraffin matrix, porous carbon and Ce (CO_3_) OH can arrange polar bonds or charges under the action of alternating electromagnetic field to attenuate the power of incident microwave, like the same kind of capacitor structure (Cao et al., 2012). Third, according to Cao’s electron hopping model (Song et al., 2009; Cao et al., 2010), electrons can absorb electromagnetic energy and migrate on the porous carbon surface, and Ce (CO_3_) OH nanoparticles can be used as a bridge for electron hopping, thus improving the conductive loss, and further converting electromagnetic energy into thermal energy through collision with the lattice (Cao et al., 2018).

As shown in Table 1, we summarize the literature on Ce (CO_3_) OH/C composites as microwave absorbing materials and Ce as filling materials reported in this work (Wang et al., 2016; Xing et al., 2017; Ge et al., 2019). Obviously, among the reported Ce filled carbon matrix composites, the prepared Ce (CO_3_) OH/C nanocomposites have good microwave absorption properties, strong absorption and thin thickness.

**TABLE 1:** Typical Ce-based composites as microwave absorbers reported in this work and recent literatures.

Samples	Matrix	RL_min_ (dB)	EAB (GHz)	Thickness (mm)	Ref
Ce (CO_3_) OH/C	Paraffin	−47.67	5.52	2.2	This work
PANI/CeO_2_ nanocomposite	Paraffin	−40.0	4.0	3.0	Xing et al. (2017)
CeO_2_/Fe_3_O_4_ composite	Paraffin	−28.9	—	7.8	Wang et al. (2016)
CeO_2_/Fe composite	Paraffin	−17.0	4.24	1.24	Ge et al. (2019)

## 4 Conclusion

In summary, a new composite material composed of porous carbon derived from polymer gel decorated with rare earth compound [Ce (CO_3_) OH] was successfully synthesized by one-pot hydrothermal method. The results show that compared with pure carbon materials, the prepared composites [Ce (CO_3_) OH/C] show significantly higher pyrroline N value, and the dispersion of the particles is better. In addition, the introduction of Ce (CO_3_) OH can improve the impedance matching of porous carbon materials, which has a significant effect on electromagnetic parameters and microwave absorption properties. When the filling ratio is 10 wt%, the prepared nanocomposites exhibit the best RL of −47.67 dB and EAB of 5.52 GHz at a thickness of only 2.2 mm. By adjusting the matching thickness from 1 mm to 3 mm, the matching thickness can be below 10 dB, covering almost the entire Ku and X-band. In addition, the possible microwave absorption mechanism of nanocomposites is proposed, which can be attributed to the synergistic effect of dipole polarization, defect polarization and interfacial polarization. In addition, the conductive loss is increased due to electron migration. Therefore, the prepared nanocomposites can be used as efficient absorbing materials in the field of electromagnetic wave absorption.

## Data Availability

The original contributions presented in the study are included in the article/Supplementary Material, further inquiries can be directed to the corresponding authors.
